# Shortened leukocyte telomere length is associated with reduced pulmonary function and greater subsequent decline in function in a sample of World Trade Center responders

**DOI:** 10.1038/s41598-019-44625-1

**Published:** 2019-05-31

**Authors:** Sean A. P. Clouston, Norman H. Edelman, Abraham Aviv, Candace Stewart, Benjamin J. Luft

**Affiliations:** 1 Program in Public Health and Associate Professor of Family, Population, and Preventive Medicine, Stony Brook University, Stony Brook, NY USA; 2 Program in Public Health, Professor of Family, Population, and Preventive Medicine, Professor of Medicine, Stony Brook University, Stony Brook, NY USA; 3 Hypertension Research Center, Professor of Pediatrics, New Jersey Medical School, Rutgers, The State University of New Jersey, Newark, NJ USA; 4 World Trade Center Health and Wellness Program, Commack, NY USA; 5 World Trade Center Health and Wellness Program and Professor of Medicine, Stony Brook University, Stony Brook, NY USA

**Keywords:** Senescence, Predictive markers, Occupational health

## Abstract

The objective of this study was to examine whether shorter leukocyte telomere length (LTL) is associated with more rapid pulmonary function decline in a longitudinal study of World Trade Center (WTC) responders. WTC responders (*N* = 284) participating in a monitoring study underwent blood sampling and were followed prospectively for spirometric outcomes. A single blood sample was taken to measure LTL using southern blotting. Outcomes included percent-predicted one-second forced expiratory volume (FEV1%), forced vital capacity (FVC%), and the FEV1/FVC ratio. In a subset, percent-predicted diffusing capacity (DLCO%) was also measured. Longitudinal modeling examined prospectively collected information over five years since blood was banked was used to examine the rate of change in pulmonary functioning over time. Severity of WTC exposure was assessed. Shorter LTL was associated with lower FEV1% and FVC% at baseline. For example, 29.9% of those with LTL <6.5 kbps had FEV1% <80% whereas only 12.4% of those with LTL ≥6.5 had FEV1% <80% (RR = 2.53, 95%CI = [1.70–3.76]). Lower DLCO% was also significantly associated with shorter LTL. Longitudinal models identified a prospective association between shorter LTL and greater yearly rates of decline in FEV1% (0.46%/year, 95%CI = [0.05–0.87]) and in the FEV1/FVC ratio (0.19%/year, 95%CI = [0.03–0.36]). There were no associations between severity of exposure and either LTL or pulmonary function. Longitudinal analyses revealed that shorter LTL, but not severity of WTC exposures, was associated with poorer pulmonary functioning and with greater subsequent decline in pulmonary functioning over time. These findings are consistent with the idea that shortened LTL may act as a biomarker for enhanced pulmonary vulnerability in the face of acute severe toxic inhalation exposures.

## Introduction

Telomeres are nucleoprotein complexes, consisting of repetition of the TTAGGG nucleotide sequences forming the end of human chromosomes, that maintain chromosomal stability and control cellular senescence^1^. Shorter leukocyte telomere length (LTL), a measure of the mean length of repeated TTAGGG sequences in leukocytes, has been linked with decreased survival in older adults^2^. Recently, one systematic review and meta-analysis of 12,595 individuals identified a consistent association between LTL and one-second forced expiratory volume (FEV1)^3^. Together, these studies have revealed associations between shorter LTL and several measures of poorer lung function and increased risk of emphysema^4–7^. Supporting this, studies have also identified correlations between intense, long-term smoking and shorter LTL^8^, though associations were weak^9^. The primary explanation given is that LTL links physical exposures with progressive disease processes via accelerated aging^10^. However, increasingly researchers have suggested that shorter LTL might also act as a biomarker of greater vulnerability to toxic exposures^11^. To date, nothing is known about the association between LTL and pulmonary function in the context of an acute but severe exposure to fine airborne particulate matter.

During the events of 9/11/2001, individuals who helped with rescue and recovery operations at the World Trade Center (WTC) were acutely exposed to a range of physical and psychological challenges including exposure to the WTC expelled dust cloud, smoke from open debris fires, and toxic dust while digging for survivors^12^. Since then, exposures at the WTC disaster site have been linked to significant reductions in percent-predicted FEV1 (FEV1%)^13^ and increased prevalence of asthma^14^, as well as a number of age-related diseases including cognitive impairment^15^ and physical limitations^16^ suggesting that WTC exposures may have caused systemic degradation resulting in more rapid aging. After the WTC events, a number of investigators have examined potential ongoing risk and resilience factors that may influence the rate of decline in pulmonary functioning including, for example, smoking and smoking cessation^17^, as well as weight gain, and bronchodilator response^18^. To date, no studies have clarified cellular predictors of deficits or declines in pulmonary functioning among WTC responders.

The objective of the current study was to understand the association between LTL and pulmonary function in the face of severe exposures to inhaled particulate matter. In addition, the ability to follow pulmonary function over time after the acute exposure gave us the unique opportunity to test whether LTL was associated with subsequent rate of decline in pulmonary functioning.

## Method

### Setting

This study represents a retrospective study of WTC responders who participated in a monitoring program at Stony Brook University (SBU). The SBU clinic monitors WTC responders residing on Long Island, NY. SBU’s population is similar in terms of exposure and age on 9/11/2001 to the general responder population^12^. Visits of responders take place at 12–18-month intervals and include a medical history, physical examination, and pulmonary function testing. Exposure assessments were done when responders were first enrolled in the clinic (between 2002–2011). Blood sampling was completed in the 2011/12 year. Pulmonary testing used in this study was completed prospectively following blood sampling.

### Sampling

Simulation power analyses suggested that a sample of 200 individuals were necessary to detect a moderate association (*r* = 0.20). Multivariable power analyses further clarified that a sample of 284 would be sufficient to identify an association between LTL and FEV1% after controlling for age. Samples were eligible for this analysis if responders’ files had complete information on exposure, age, sex, and had valid pulmonary functioning tests and usable blood (*N* = 284).

To examine the association between LTL and change in pulmonary functioning over time, LTL data were merged with follow-up information collected in the years since LTL was collected. Spirometry data was collected on these responders a total of 1,355 times for an average of 4.8 pulmonary follow-ups collected since blood was retrieved for LTL assays.

### Ethics approval and consent to participate

This study was reviewed by the Institutional review board (CORIHS: IRBNET# 604113). Responders provided written informed consent.

## Ethics, Consent, and Permissions

WTC responders in this study provided written informed consent. The Stony Brook Institutional Review Board approved this study (CORIHS#604113). All experimental conditions were performed in accordance with relevant guidelines and regulations.

## Measures

### Pulmonary functioning

Spirometry was performed, as part of the parent surveillance program (training and data quality are overseen by CDC staff), by trained technicians before and after administration of an inhaled bronchodilator using an EasyOne spirometer [Medical Technologies USA] and associated race/gender/age-specific predicted values^19^. Percent-predicted values of FEV1 (FEV1%) and forced vital capacity (FVC%) were reported, as was the FEV1/FVC ratio (in percent). Additionally, the bronchodilator response (BDR) for each responder using the post bronchodilator FEV1 expressed as a percentage of the pre-bronchodilator FEV1^20^. Pulmonary functioning measures underwent validation and were deemed acceptable if there were no early procedural terminations, variable efforts, leaks, obstructed mouthpieces, or other physical artefacts. In a subset of subjects (*n* = 37) single breath diffusing capacity was reported as percent-predicted (DLCO%). For descriptive analyses, low FEV1% was defined as FEV1% <80%.

Medical histories were examined for diagnoses of obstructive airways disease, upper respiratory disease, asthma, pulmonary fibrosis, and cancer of the lung and bronchus. None of the subjects carried diagnoses of cancer or pulmonary fibrosis. However, since pulmonary fibrosis was considered to be of especial significance to this cohort, a pulmonary specialist [NHE] reviewed the reports of chest radiographs, which were available for 89.8% of the sample, for findings that might be considered consistent with early fibrosis.

### Leukocyte telomere length

Many studies have measured LTL using a PCR-based method, which quantifies telomere DNA content as the ratio of telomeric to single copy gene PCR product^21^. However, this is not readily transformed into their units of measurement (kbps)^22^. This study therefore utilized southern blotting of terminal restriction fragments, a method that has been shown to provide the most specific and reliable measure of LTL available^23^. LTL was expressed in kilobase pairs (kbps). For descriptive purposes, LTL was split into high *versus* low groups using an age-adjusted cutoff matching the lowest decile (cutoff = 6.5 kbps) from a population sample using the same method to measure LTL^24^.

### Other variables

WTC exposure severity was assessed during the enrollment visit using a structured history. An exposure severity index was created using a previously validated measure that utilized a Delphi technique to weight a range of pulmonary exposure measures experienced at the WTC both on 9/11/2001 and in the following months^25^.

Occupation was dichotomized into law enforcement (including New York Police Department, Federal Bureau of Investigations, other local police, and security workers), and non-traditional responders (including predominantly construction and utility workers, but also reporters, therapists, medics, and other volunteers). Smoking status was assessed at baseline and characterized responders as current, former, or never smokers.

### Statistical analysis

Descriptive sample statistics used T-tests and proportions tests in bivariate analyses linking covariates. T-tests were also used to compare key study variables in those who were selected for LTL assays compared to those in the population who were not. Pearson’s correlation coefficients were reported to show unadjusted associations between LTL and multiple measures of pulmonary functioning.

Longitudinal data were analyzed utilizing multilevel modeling to examine FEV1%, FVC%, and the FEV/FVC ratio^26^. Models make use of all available data to provide reliable estimates but assume that data are multivariate-normal, and that data are missing at random due to processes that are captured by fixed or random effects components in the modeling process^26^. In addition to covariates included in the model, longitudinal analyses adjust for individual-level time-invariant differences in pulmonary function, heteroskedasticity over time that is common in longitudinal modeling, and for regression toward the mean that occurs when individuals are monitored over time using the same test^27^. Analyses were separated into predictors of baseline differences in pulmonary function and predictors of change over time in pulmonary function. Beta-coefficients (B), standard errors (SE), and p-values derived from t-tests were reported. A two-tailed alpha = 0.05 was used to determine statistical significance. Due to potential multiple testing biases, the impact of adjusting alpha for false discovery rates was examined^28^; since this resulted in no change to substantive conclusions, we reported nominal p-values in all analyses. Adjusted R^2^ were reported and R^2^-change was used to examine changes to model fit. All analyses were completed using Stata 14/IC [StataCorp].

### Role of the funding source

The funders played no role in: study design; data collection, analysis, or interpretation; developing the report for publication; or the decision to submit this paper for publication. The corresponding author had full access to all data in the study and had final responsibility for the decision to submit this study for publication.

## Results

### Sample characteristics

On average, the sample was made up of male law enforcement responders, half of whom arrived on 9/11/2001. The average LTL was 6.9 kbps. Most responders had at least some college education, and only one-eighth of those sampled currently smoked. The average responder had FEV1/FVC of 79.72%, and FEV1% was 92.82%. More than one-quarter of the sample had diagnosed asthma, approximately one-quarter had FEV1% <80%. Only a small number (*n* = 8) had radiographic signs suggestive of early pulmonary fibrosis Table 1.

**Table 1:** Sample characteristics.

Characteristic	Mean (SD)
LTL, kbps	6.94 (0.62)
Age, years	49.66 (8.87)
WTC Exposure Severity	18.61 (6.14)
FEV1%	92.82 (14.46)
FVC%	106.44 (17.15)
FEV1/FVC	79.72 (5.84)
DLCO%*	84.64 (20.74)
Bronchodilator Response (%)	−1.41 (3.40)
**Characteristic**	**N (%)**
Asthma	66 (27.7)
FEV1% <80%	69 (24.3)
Radiographic Evidence of Early Pulmonary Fibrosis^†^	8 (3.1)
Smoking Status	
Never Smoker	185 (65.2)
Former Smoker	66 (23.2)
Current Smoker	33 (11.6)
Female Sex	14 (4.9)

Note: LTL: Leukocyte telomere length; FEV1%: Percent predicted one-second forced expiratory volume; FVC%: percent-predicted forced vital capacity; FEV1/FVC: the ratio (in percent) of one-second forced expiratory volume divided by forced vital capacity; WTC: World Trade Center; DLCO%: percent-predicted diffusing capacity of the lung for carbon monoxide. *37 responders had valid DLCO%. ^†^255 responders had x-ray information necessary to derive this measure.

### Sample differences versus the population

Comparing the sample of those whose blood was assayed for LTL to those without the LTL measure who were followed up in the same year (*N* = 4,290) we find that those with LTL data were not older (*P* = 0.595) and did not differ in terms of FVC% (*P* = 0.420), FEV1% (*P* = 0.714), or occupation (*P* = 0.484) compared to those who were not selected for the study.

### Characteristics of those with shorter LTL

Sample stratification into those with LTL <6.5 kbps (N = 67, 23.6% of the sample of 284) *versus* those with LTL ≥6.5 kbps revealed that responders with short LTL were older, and had reduced FEV1%, FVC%, and DLCO% than those with longer LTL. For example, 44.8% of those with short LTL had FEV1% <80%, whereas only 18.0% of those with LTL ≥6.5 had FEV1% <80% (RR = 2.49, 95%C.I. = [1.69–3.68]) Table 2.

**Table 2:** Sample characteristics separated by those with and without short LTL.

Characteristics	LTL < 6.5	LTL ≥ 6.50		
	Mean (SD)	Mean (SD)	Diff.	P
Age, years	53.34 (11.27)	48.53 (7.67)	−4.81	0.001
WTC Exposure Severity	18.65 (5.75)	18.60 (6.26)	−0.05	0.955
FEV1%	82.25 (17.72)	91.05 (13.66)	8.08	<0.001
FVC%	98.74 (20.67)	108.82 (15.19)	10.09	<0.001
FEV1/FVC%	79.07 (6.08)	79.93 (5.77)	0.86	0.291
DLCO%*	81.72 (11.18)	95.37 (18.12)	13.65	0.004
Bronchodilator Response (%)	−1.69 (3.21)	−1.32 (3.46)	0.38	0.429
**Categorical Variable**	**N (%)**	**N (%)**	**(95% CI)**	**P**
Asthma	25 (37.31)	60 (27.65)	1.39 (0.91–2.13)	0.132
FEV1% <80%	30 (44.78)	39 (17.97)	2.53 (1.70–3.76)	<0.001
Radiographic Evidence of Early Pulmonary Fibrosis^†^	2 (3.28)	6 (3.09)	1.04 (0.31–3.55)	0.942
Smoking Status				
Never Smoker	39 (58.21)	146 (67.28)	1.00	
Former Smoker	20 (29.85)	46 (21.2)	1.44 (0.91–2.28) 1.15	0.130
Current Smoker	8 (11.94)	25 (11.52)	(0.59–2.23) 1.22	0.694
Female Sex	4 (5.97)	10 (4.61)	(0.52–2.88)	0.653

Note: LTL: Leukocyte telomere length; FEV1: One-second Forced Expiratory Volume; FVC: Forced Vital Capacity; FEV1%: Percent predicted FEV1; WTC: World Trade Center; DLCO: Diffusing capacity of the lung for carbon monoxide. *36 responders had valid %-Pred. DLCO. ^†^255 responders had x-ray information necessary to derive this measure. P-values examining differences between responders with shorter and longer LTL were derived from Student’s T-tests for continuous measures and from nonparametric trend tests for categorical measures.

### LTL and change in pulmonary functioning over time

Longitudinal analyses examining rate of change in pulmonary functioning over time (Table 3) revealed that the average responder experienced significant reductions in FEV1% (−5.62% per year) and in FEV1/FVC (−3.28% per year) during the study period. Analyses further revealed that longer LTL was associated with slower rates of decline in FEV1% and in the FEV1/FVC ratio. In contrast, WTC exposure severity was associated with a small positive effect on longitudinal change in pulmonary functioning potentially indicative of pulmonary improvement after severe exposure. Furthermore, smoking status at baseline was associated with longitudinal improvement in FEV1%, FVC%, and FEV1/FVC ratios, potentially indicative of benefits to smoking cessation occurring during the study period Table 3.

**Table 3:** Multivariable associations linking leukocyte telomere length and longitudinal rate of change in pulmonary functioning

Characteristic	FEV1%			FVC%			FEV1/FVC%		
	B	SE	P	B	SE	P	B	SE	P
LTL	0.46	0.21	0.028	0.31	0.24	0.191	0.19	0.08	0.023
WTC Exposure Severity	0.06	0.02	0.008	0.07	0.03	0.007	0.00	0.01	0.902
Age in years	0.02	0.01	0.244	−0.03	0.02	0.042	0.05	0.01	<0.001
Never Smoker	1.00			1.00			1.00		
Former Smoker	−0.22	0.30	0.475	−0.28	0.35	0.422	0.02	0.13	0.897
Current Smoker	1.06	0.39	0.007	0.94	0.45	0.037	0.33	0.16	0.043
Slope	−5.62	1.90	0.003	−2.84	2.17	0.190	−3.28	0.77	<0.001
Random Slope (SD)	0.79	0.10		0.72	0.14		0.23	0.03	

Note: LTL: Leukocyte telomere length; FEV1: One-second Forced Expiratory Volume; FVC: Forced Vital Capacity; FEV1%: Percent predicted FEV1; WTC: World Trade Center; B: Beta coefficient; SE: Standard error; P: p-values derived from two-tailed t-tests. *Note that only longitudinal associations were shown.

## Discussion

Decreased leukocyte telomere length (LTL), which has been postulated to be a cellular marker of systemic aging^29^, has been previously linked to poorer pulmonary functioning. However, to the authors’ knowledge, studies have not yet documented associations between pulmonary function and LTL after acute but severe inhalations of airborne particulate matter. Nor have prior studies examined the prospective association between LTL changes in pulmonary functioning over time, or the distribution of LTL among responders to the tragic events at the WTC on 9/11/2001. This study examined linkages between LTL and pulmonary functioning in a sample of men and women who participated in the WTC response efforts during and after 9/11/2001. The current study then documented that responders with shorter LTL (LTL ≤6.5 kbps) were at heightened risk of having low pulmonary functioning. Longitudinal analyses further revealed that individuals with longer LTL had slower rates of change in FEV1% and FEV1/FVC over time. Yet, more severe exposures at the WTC site was not predictive of shorter LTL or decreased pulmonary functioning. Analyses supported the view that shorter LTL may predict more rapid declines in pulmonary functioning.

### Implications

The present results appear to be relatively generalizable to the WTC responder cohort. For example, prior work has estimated that WTC exposures are associated with a 12% decrease in FEV1%^13^, while other work has identified associations between WTC exposure severity and increased risk of asthma^14^. The current study documented a similar 11% lower FEV1% among sampled responders, similar overall levels of asthma to those reported in the greater WTC responder population^14^, and also replicated associations between age and both LTL and FEV1% reported in the literature. Additionally, the present study extends this line of research by showing that shorter LTL was correlated with reduced pulmonary function after the WTC acute toxic inhalational exposure and by identifying for the first time a prospective association between shorter LTL and more rapid declines in FEV1% and FEV1/FVC over time.

Examining indicators of pulmonary functioning revealed associations between LTL and FEV1%, FVC%, and DLCO% but not with the FEV1/FVC ratio when first measured, measures of airways reactivity or the incidence of reported asthma, suggesting that the lung damage related to LTL was more likely to be of the parenchyma than the airways. However, we note that none of the radiographic reports indicated clear interstitial lung abnormalities. Critically, in this study longitudinal analyses revealed that shorter LTL was associated with more rapid declines in FEV1% and FEV1/FVC ratios in the years following the acute exposure. Accordingly, the present findings are consistent with the idea that LTL shortening served as a vulnerability marker for progressive pulmonary abnormalities, perhaps including airways, following exposure to an acute inhalation of dust at the WTC. However, there is a possibility that more rapid declines may simply be related to subclinical inflammatory processes ongoing among those with reduced pulmonary functioning^30^. Thus, these results cannot rule out the possibility that declines in FEV1% co-occur with declines in LTL. Future work will be needed to examine the extent to which domains of changes in LTL co-occur with or instead predict changes in pulmonary functioning.

A major goal of aging research is to identify mechanisms and biomarkers of aging and of environmental exposures that might cause more rapid aging^10^. This study examined LTL and its associations with pulmonary functioning approximately one decade after severe exposures undergone during the WTC disaster. The findings supported prior research in identifying associations between age and LTL at a degree comparable to results in the general population^31^, but smaller than those reported in the Framingham study^9^. The results from this study were consistent with the interpretation that shorter LTL may be a marker of biologic vulnerability^11^ and of increased vulnerability to decline in pulmonary functioning in the context of a severe acute pulmonary exposure.

This study found inconsistent associations between exposure severity and pulmonary functioning in this cohort. A lack of association between LTL and exposure severity may be due to limited power in this study to detect differences. Similarly, the lack of association between smoking status and pulmonary functioning measures may stem from the small number of current smokers. Future work should identify stronger metrics of WTC pulmonary exposures in order to clarify results found here. Specifically, to our knowledge no WTC exposure measure has yet integrated specific WTC activities. Inclusion of specific activities may help in future analyses to better understand pulmonary reactions to WTC exposures.

### Limitations

This study was the first to examine associations between WTC exposures, LTL, and longitudinal changes in pulmonary functioning. Results are limited to participants whose biological data were successfully collected and assayed. Responders whose LTL were screened do not differ from other responders in terms of age, FEV1% or FVC% but it is unclear to what extend LTL in this sample is representative of WTC responders in general. The small sample size in this study resulted in a limited power to detect racial/ethnic differences in either LTL or in the outcomes of interest. Further research is needed with a larger sample size to determine the role of race/ethnicity in moderating these results. As this study lacked longitudinal information on LTL, this study cannot discern whether declines in FEV1% are indicative of a shared systemic degradation that may also influence LTL. Further longitudinal work should examine whether rates of decline in LTL are concomitant with changes in pulmonary functioning in order to improve our understanding of this process. While pulmonary functioning tests are reliable, there remains variability in their rates of change over time that may be due to biological factors or measurement error. Further work using more specific measures indicative of particular disease processes may help to clarify mechanisms linking pulmonary functioning with LTL. Finally, while exposure severity in this cohort was measured in a consistent and detailed manner, the generalizability of effects of pulmonary exposures in this cohort to other pulmonary exposures is limited.

## Conclusions

Data from this cohort of WTC responders provide a resource with which to understand long-term effects of acute toxic inhalational pulmonary exposures. These data show that poorer pulmonary functioning at first test, suggestive of parenchymal abnormalities, was associated with shorter LTL, Furthermore the data show that shorter LTL was associated with greater declines in pulmonary function with time following the acute exposure. Considering the lack of relationship between LTL and degree of toxic exposure these findings are consistent with the idea that shortened LTL acted as a vulnerability factor for pulmonary damage although we cannot rule out that it was a result of the exposure itself.

## Data Availability

Data from the monitoring programs is publicly available through an independent application process at Icahn School of Medicine at Mount Sinai (https://icahn.mssm.edu/about/departments/environmental-public-health/research/wtc-data-center). Deidentified data derived from this study can also be made available upon request by the corresponding author.
